# ‘Dragon's Den’: Gamifying Handover Skills Teaching

**DOI:** 10.1111/tct.70338

**Published:** 2026-01-25

**Authors:** Mary Catherine Mina, Mu'Azzamah Ahmad, Janet Skinner

**Affiliations:** ^1^ Clinical Skills Centre, Edinburgh BioQuarter Chancellor's Building, Edinburgh Medical School University of Edinburgh Edinburgh UK

**Keywords:** communication, gamification, handover, innovation, medical education, referrals

## Abstract

**Background:**

Effective communication during patient handovers is critical to ensuring patient safety. While previous teaching methods locally have been successful, they have typically relied on substantial faculty input and resources. Drawing inspiration from contemporary approaches to small group learning, this study explores the impact of a gamified clinical simulation—delivered through an interactive, ‘Dragon’s Den’‐style format—on medical students’ self‐reported confidence and preparedness in handover and referral skills.

**Approach:**

A 90‐min workshop was developed incorporating a ‘Dragon’s Den’‐inspired activity. Students assumed the role of ‘investors’, in teams, using a structured checklist to evaluate pre‐recorded video handovers. This was followed by paired practice of referrals with fictional clinical scenarios, during which students gave and received peer feedback, using the same checklist. Paired pre‐ and postsession responses were collected via Wooclap to assess changes in self‐reported confidence and preparedness. An anonymous free‐text feedback form was also distributed following the session.

**Evaluation:**

Quantitative analysis revealed a statistically significant improvement in both confidence and preparedness. Thematic analysis of qualitative feedback highlighted that students found the session engaging, relevant, and valuable; however, some expressed a desire for additional resources and suggested the session may be beneficial if scheduled earlier in the curriculum.

**Implications:**

This study supports the use of gamified clinical simulation as an effective method for teaching handover skills. The peer‐led, experiential format offers a scalable, low‐resource approach that aligns well with the constraints of modern medical curricula. Moreover, this model carries potential for broader application in wider educational settings.

## 1 Background

There is a growing demand to better prepare medical students for clinical practice, particularly considering reduced placement opportunities caused by increasing medical school intake across the United Kingdom [1]. Furthermore, graduates often report feeling unprepared for practice—specifically when it comes to challenging communication tasks [2]. One area is delivery of handovers and referrals.

The General Medical Council (GMC) highlights the importance of medical graduates being competent in the tasks of delegation and referral, requiring a sound understanding of the patient's clinical context as well as the ability to communicate clearly with colleagues [3]. In addition, poor communication between healthcare professionals is widely recognised as a contributor to adverse patient outcomes [4, 5].

To address this, final‐year students at Edinburgh Medical School attended a referral simulation session with the aim of boosting their confidence and competence [6]. While successful, the format was resource‐intensive, requiring significant faculty involvement.

In response to this, and inspired by new methods of engagement through innovative small group learning, we embarked on a project to develop a renewed workshop to address these challenges.

Gamification, defined as ‘the use of game design elements in non‐game contexts’, is an emerging method of teaching that is gaining traction in medical education [7]. This form of teaching is a powerful tool that has been shown to increase student enjoyment, motivation and engagement in learning tasks [8]. In addition, gamification is shown to encourage collaboration among students by offering opportunities to work as a team; a core skill necessary for success in a career in healthcare [9]. To our knowledge, there are no published articles describing this method to teach handover and referral skills to undergraduate medical students.

This project aimed to evaluate the impact of using gamified clinical simulation as a novel teaching approach on two self‐reported student outcomes: confidence in delivering a handover and preparedness in using a standardised framework to structure a handover.

*Gamification is shown to encourage collaboration among students by offering opportunities to work as a team*.

## 2 Approach

**FIGURE 1:**
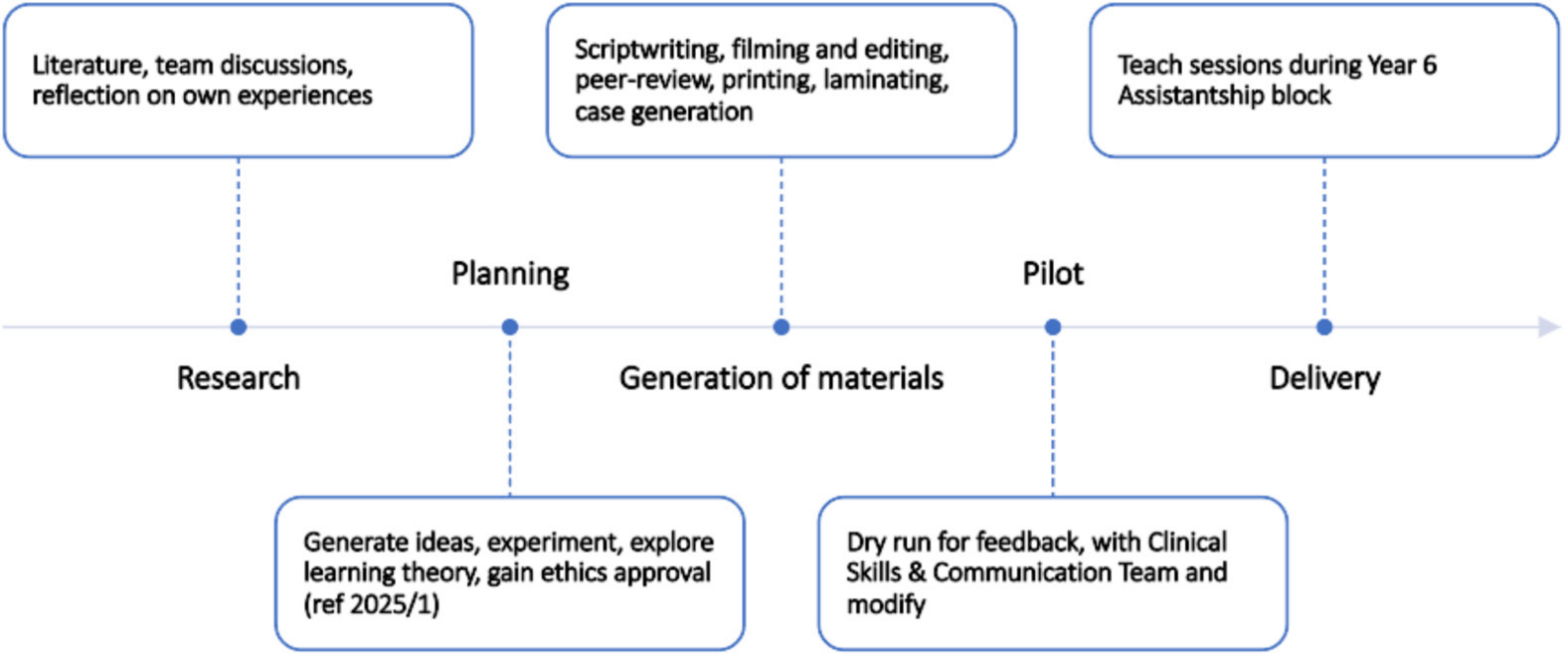
Timeline of session development.

The practical workshop was developed over 6 months, as illustrated in Figure 1. The process began with a comprehensive literature review, alongside critical reflection on clinical experiences of team members relating to handovers and referrals in their corresponding healthcare environments. These insights informed the creation of a relevant, collaborative teaching session.

Constructivism, social learning and experiential learning theories informed the design of our chosen interactive activities that encouraged students to collaborate and learn through doing, observing, reflecting and imitation [10, 11, 12] (see Table 1).

**TABLE 1:** Lesson plan design informed by learning theories.

Timings	Learning theory	Key concept	Application
Activity 1 Think‐pair‐share (10 min)	Constructivist learning (Vygotsky)	Building knowledge through collaborations and reflection to boost confidence and preparedness	Group discussions, snowballing activities and peer collaboration
Activity 2 Dragon's Den (30 min)	Social learning (Bandura)	Learning though observation and imitation to improve preparedness	Watching 3 pre‐recorded handovers and group feedback
Activity 3 Peer practice (25 min)	Experiential learnings (Kolb)	Learning by doing and reflecting to improve confidence	Hands‐on SBAR practice, feedback loops and debriefing

The session started with a Think‐Pair‐Share activity, designed to encourage reflection and discussion around prior knowledge as well as introducing the newly adapted SBAR‐D structure for handovers (Situation, Background, Assessment, Recommendation, Decision) [13] as outlined in Photograph 1. Our main activity was influenced by the concept of gamification in learning, with inspiration drawn from the popular television programme ‘Dragon’s Den’ [14]. This programme features opportunities for hopeful entrepreneurs to pitch their proposed business ideas to a panel of investors called ‘Dragons’ who will then decide if the idea is worthy of investment. We developed a series of video examples depicting different handover and referral scenarios, such as handover of a patient at shift changeover. In teams of four, students took on the role of ‘Dragons’, using structured checklists to evaluate and critique each case, as demonstrated in Photograph 2. If a referral ‘pitch’ was compelling enough, they could ‘invest’ using prop money provided as part of the game. Learning points from these discussions were then applied in a follow‐up activity ‘Peer‐Practice’ where students made referrals to each other using fictional patient cases, such as requesting advice from Microbiology. Provided materials included patient notes, NEWS charts, blood results and fluid/drug charts. Students then received peer feedback using the same evaluation checklist used in the ‘Dragon's Den’ Activity.

**PHOTOGRAPH 1:**
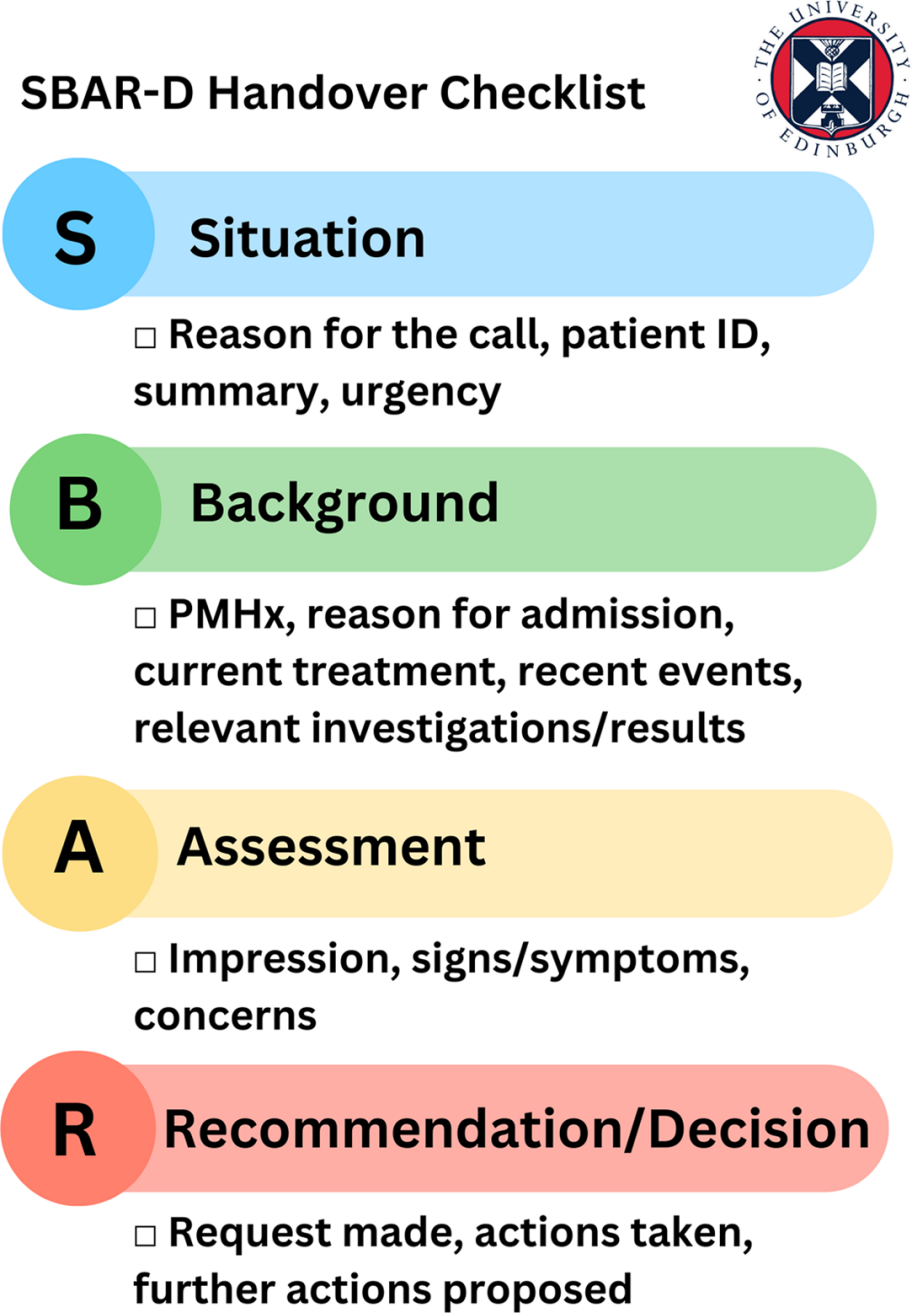
Overview of SBAR‐D structure.

**PHOTOGRAPH 2:**
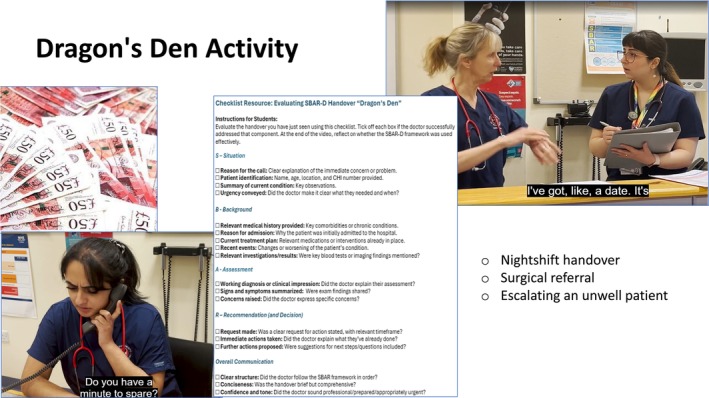
Learning materials used for the ‘Dragon's Den’ activity.

*Students took on the role of ‘Dragons’, using structured checklists to evaluate and critique each case*.

The workshop was delivered during the ‘Assistantship’ module of the final year MBChB programme as 90‐min, face‐to‐face sessions with a maximum of 20 participants. We ran 16 sessions from March to May 2025. Each session was facilitated by up to three staff members, including clinical education fellows and doctors at varying levels of training, who shared their own experiences and practical advice.

**FIGURE 2:**
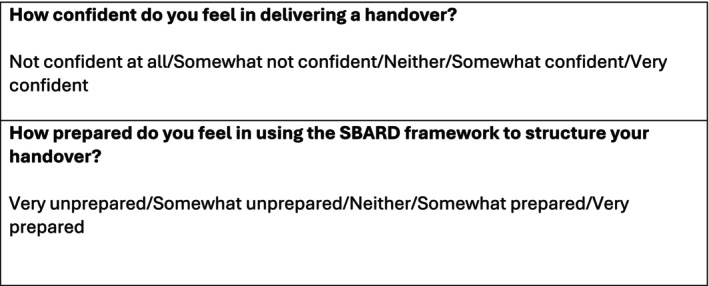
Survey questions asked via Wooclap.

Data were collected using a mixed‐method evaluation approach. Students anonymously completed paired pre‐ and postsession surveys through the interactive platform ‘Wooclap’, responding via their phones to Likert‐scale questions assessing self‐reported confidence in delivering a handover and preparedness in using the SBAR‐D framework (see Figure 2). At the end of the session, a brief written feedback form was sent out. Quantitative data were analysed using SPSS. A Shapiro–Wilk test indicated that the data were not normally distributed; therefore, Wilcoxon's signed‐rank test was used to assess changes between paired responses. Qualitative feedback was analysed thematically using NVivo (version 14.23.2).

Ethical approval for this study was granted by the Medical Education Research Ethics Committee (2025/1).

## 3 Evaluation

### 3.1 Quantitative Data

Two hundred sixty‐seven students attended the sessions, with 142 paired responses in total (53% response rate).

**FIGURE 3:**
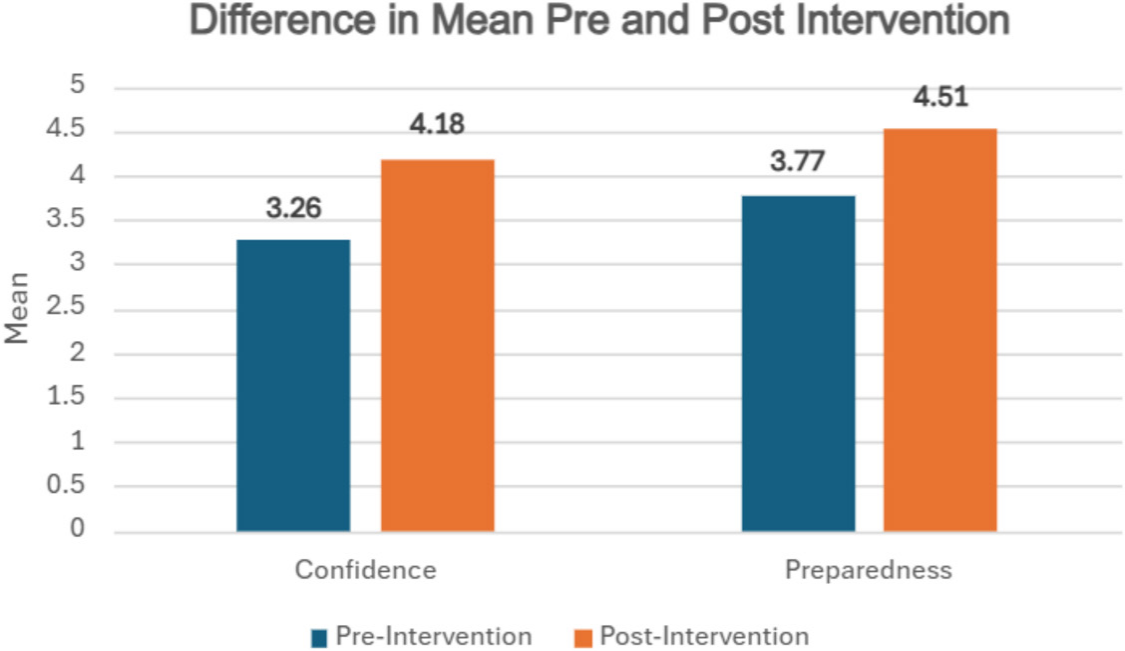
Bar Graph demonstrating Difference in Means Pre‐ and Post‐ Intervention.

Results demonstrate statistically significant improvement (*p* < 0.001) in both confidence in delivering a handover and preparedness in using SBAR‐D to structure their handover. Wilcoxon signed‐rank tests demonstrate an increase in confidence from 3.26 to 4.18 (Δ = 0.92), representing a 28% relative increase, with a large effect size (*r* = 0.72). Preparedness improved from 3.77 to 4.51 (Δ = 0.74), representing a 20% increase, also with a large effect (*r* = 0.71) (see Figure 3).

*Results demonstrate statistically significant improvement (p < 0.001) in both confidence in delivering a handover and preparedness in using SBAR‐D*.

### 3.2 Qualitative Data

There were 196 responses to the brief free‐text feedback form (73% response rate). Thematic analysis of the responses generated the following themes summarised below (see Table 2):

**TABLE 2:** Results of thematic analysis.

Theme	Summary	Quotes
Interactivity and engagement	Students enjoyed learning through interactive activities. Collaboration during the discussions and critiques of the handovers encouraged engagement, making learning fun.	‘Working together in groups and interacting with teacher and peers to keep it stimulating’ ‘Very interactive, the dragon's den idea was really good’ ‘I enjoyed critiquing other handovers and understanding what makes one good/not so good’ ‘It made learning SBAR really fun’
Relevant and valuable	Participants valued practice in a safe space. The scenarios were appreciated for their content and realism, and the students valued the lived experiences shared by the facilitators.	‘Thank you for not making us do handovers in front of the whole group—I learnt so much without feeling fear the whole time!!’ ‘The scenarios were “super detailed and helpful”’ ‘Actually, getting a very realistic quantity of information to go through’ ‘Getting useful tips from the facilitators’
Time management	Most students wanted more practice time with a consensus that this was the most valuable exercise. Some ideas were suggested on how to manage this.	‘Much more time devoted to peer practice cases which were very useful and the highest yield’ ‘I think spending a while going over SBAR‐D etc. might have been quite a bit of revision, I think it would be more helpful to spend more time doing more practical cases (instead of just 20mins at the end!)’ ‘Slightly less time on dragons' den as it was fun but less helpful than practicing. Then could have more time to practice’
More resources	Students expressed an interest in having more resources: more video examples, new scenarios, further discussions and specialty‐specific information and advice.	‘Maybe more examples of the videos‐ helps show the variety and maybe different errors you could make in a sbar’ ‘More cases given numbers per group’ ‘It would be helpful if we all got more turns to do different clinical scenarios each as calling different specialties like microbiology, surgery, other HCPs, etc. leads to different type of content you'd include in the SBARD’ ‘I think at this stage of y6 we are familiar with SBAR formats. I think it would be beneficial to hear from clinicians who regularly receive referrals I.e., Radiology, Surgery, Medicine, ICU and hear from their perspective what they look for when receiving referrals’
Timetabling	Students highlighted the desire to have this session delivered earlier in their education to allow for a chance to implement the knowledge received in their practice.	‘Really good practical advice, more of the same would be great with an introduction session at the beginning of year 6 would be beneficial’ ‘Maybe have it earlier on in the block to give more time to put it to practice in assistantship’ ‘Believe this should be done when students start placement in 4th year too’

## 4 Implications

This project outlines the development and implementation of a gamified interactive workshop designed to improve confidence and preparedness of upcoming graduate doctors in their skills of handovers and referrals.

The high response rate to the feedback form increases the reliability of our results. Key strengths of this initiative include the use of experiential learning, enabling students to practice clinical communication in a supportive environment, reflect on their performance and engage in peer feedback. The use of fictional patient scenarios provided realistic context with clear applicability to everyday clinical practice, enhancing relevance and learner engagement. Gamification elements further promoted active participation and critical thinking, facilitating the development of knowledge in a dynamic and enjoyable team‐based format [8].

Based on our experience, we argue that gamified clinical simulation offers a scalable approach to preparing students for future clinical roles. This model enables the efficient delivery of high‐quality teaching to large student cohorts within a compressed time frame. Crucially, it aligns with GMC outcomes [3] while responding to the growing challenge of constrained clinical placement availability.

This workshop model lends itself well to sustainability, using low‐cost resources and minimal faculty involvement due to the incorporation of structured checklists for feedback. Although preparation of the materials—such as laminated documents and short, scripted videos—was time‐intensive, their reusability contributes to the session's longevity, as advocated by Singhal et al. [8].

*This workshop model lends itself well to sustainability, using low‐cost resources and minimal faculty involvement*.

The format could be adapted for interprofessional and postgraduate education by expanding the scope of referral scenarios beyond medicine and increasing complexity. The approach could also be used to explore challenging referral and handover situations, including dealing with incivility.

An unanticipated finding was students' strong preference for peer‐led, independent learning. While faculty members provided opportunities for direct observation and feedback, students favoured autonomous engagement with the materials, mirroring the self‐directed nature of clinical placements. This brings forth the importance of peer feedback as a meaningful skill for learning—a point supported by Cushing et al. [15], whose study found that most students preferred receiving feedback from peers rather than tutors. This raises important questions about the workshop's current placement within the curriculum with some students suggesting introducing it earlier in their training, however: Would learners in earlier years possess the same readiness for practice to engage with the session independently and effectively?

*Students favoured autonomous engagement with the materials, mirroring the self‐directed nature of clinical placements*.

Despite the success of this workshop, limitations of this evaluation include reliance on self‐reported confidence as an indicator of competence, which may not accurately reflect actual skill acquisition. Although objective measures of improved handover performance would have strengthened our evaluation, this was not feasible due to time and resource constraints but could be explored in the future. Additionally, we did not include assessment of long‐term retention or the impact on clinical performance. Moreover, responses pre‐ and postsession were dependent on mobile internet connection using a QR code, which may reflect the reduced response rate during the session compared to postsession feedback. Future research should include longitudinal follow‐up during early postgraduate training to evaluate the lasting effects on clinical readiness and performance.

This project demonstrates the effectiveness of an interactive, gamified simulation approach to teaching handover skills to medical students. The intervention resulted in statistically significant short‐term improvements in students' confidence and perceived preparedness for clinical communication during handovers and referrals. We intend to refine the workshop based on participant feedback, with particular attention to optimising the balance and timing of activities to sustain engagement and suggest further integration of authentic specialty‐specific case material.

These findings also reinforce the value of experiential, peer‐led learning in the development of communication skills. We suggest that structured, student‐centred approaches such as ours can be a valuable addition to undergraduate medical education, particularly in addressing core competencies within increasingly time‐pressured curricula but could also be beneficial in other contexts.

## Author Contributions

**Mary Catherine Mina:** conceptualization, investigation, writing – original draft, methodology, validation, visualization, writing – review and editing, formal analysis, project administration, data curation, resources. **Mu'Azzamah Ahmad:** conceptualization, investigation, writing – original draft, methodology, validation, visualization, writing – review and editing, formal analysis, project administration, data curation, resources. **Janet Skinner:** conceptualization, writing – review and editing, supervision.

## Funding

The authors have nothing to report.

## Ethics Statement

Ethical approval was sought from the Edinburgh Medical School Medical Education Unit Ethics Committee, reference number 2025/1.

## Conflicts of Interest

The authors declare no conflicts of interest.

## Data Availability

The data that support the findings of this study are available from the corresponding author upon reasonable request.
